# Mapping of Adaptive Traits Enabled by a High-Density Linkage Map for Lake Trout

**DOI:** 10.1534/g3.120.401184

**Published:** 2020-04-13

**Authors:** Seth R. Smith, Stephen J. Amish, Louis Bernatchez, Jeremy Le Luyer, Chris C. Wilson, Olivia Boeberitz, Gordon Luikart, Kim T. Scribner

**Affiliations:** * Conservation Genomics Group; Wildlife Biology Program, University of Montana, Missoula, MT,; † Flathead Lake Biological Station, Division of Biological Sciences, University of Montana Polson, MT,; ‡ Department of Integrative Biology, Michigan State University, East Lansing MI,; § Institut de Biologie Intégrative et des Systèmes, Université Laval, Québec, Canada,; ** Ifremer, IRD, Institut Louis‐Malardé, Univ Polynésie française, EIO, F‐98719 Taravao, Tahiti, Polynésie française, France,; †† Ontario Ministry of Natural Resources and Forestry, Peterborough, Ontario, Canada; ‡‡ Department of Fisheries and Wildlife, Michigan State University, East Lansing MI

**Keywords:** Linkage map, Salvelinus, QTL, RAD, genomics, lake trout

## Abstract

Understanding the genomic basis of adaptative intraspecific phenotypic variation is a central goal in conservation genetics and evolutionary biology. Lake trout (*Salvelinus namaycush*) are an excellent species for addressing the genetic basis for adaptive variation because they express a striking degree of ecophenotypic variation across their range; however, necessary genomic resources are lacking. Here we utilize recently-developed analytical methods and sequencing technologies to (1) construct a high-density linkage and centromere map for lake trout, (2) identify loci underlying variation in traits that differentiate lake trout ecophenotypes and populations, (3) determine the location of the lake trout sex determination locus, and (4) identify chromosomal homologies between lake trout and other salmonids of varying divergence. The resulting linkage map contains 15,740 single nucleotide polymorphisms (SNPs) mapped to 42 linkage groups, likely representing the 42 lake trout chromosomes. Female and male linkage group lengths ranged from 43.07 to 134.64 centimorgans, and 1.97 to 92.87 centimorgans, respectively. We improved the map by determining coordinates for 41 of 42 centromeres, resulting in a map with 8 metacentric chromosomes and 34 acrocentric or telocentric chromosomes. We use the map to localize the sex determination locus and multiple quantitative trait loci (QTL) associated with intraspecific phenotypic divergence including traits related to growth and body condition, patterns of skin pigmentation, and two composite geomorphometric variables quantifying body shape. Two QTL for the presence of vermiculations and spots mapped with high certainty to an arm of linkage group Sna3, growth related traits mapped to two QTL on linkage groups Sna1 and Sna12, and putative body shape QTL were detected on six separate linkage groups. The sex determination locus was mapped to Sna4 with high confidence. Synteny analysis revealed that lake trout and congener Arctic char (*Salvelinus alpinus*) are likely differentiated by three or four chromosomal fissions, possibly one chromosomal fusion, and 6 or more large inversions. Combining centromere mapping information with putative inversion coordinates revealed that the majority of detected inversions differentiating lake trout from other salmonids are pericentric and located on acrocentric and telocentric linkage groups. Our results suggest that speciation and adaptive divergence within the genus *Salvelinus* may have been associated with multiple pericentric inversions occurring primarily on acrocentric and telocentric chromosomes. The linkage map presented here will be a critical resource for advancing conservation oriented genomic research on lake trout and exploring chromosomal evolution within and between salmonid species.

Maintaining adaptive phenotypic diversity is a central tenet of conservation biology. In many taxa, diversity is produced through selective pressures that favor reduced intraspecific competition and trophic specialization (Skulason and Smith 1995; Robinson and Schluter 2000; Whiteley 2007). The evolution of trophically specialized morphotypes has been observed in multiple fish species including Arctic char (Snorrason *et al.* 1994), lake trout (Eshenroder 2008; Muir *et al.* 2016), multiple coregonid species (Lu and Bernatchez 1999; Thomas *et al.* 2019), and African cichlids (Rüber *et al.* 1999), and represents an important pathway by which phenotypic diversity is generated and maintained in nature (Pfennig and Pfennig 2012). Intraspecific diversity can promote community and ecosystem stability (Schindler *et al.* 2010); however, the genomic basis for this variation is often poorly understood for non-model species. Advancement of our understanding is largely limited by a lack of genomic resources.

Lake trout (*Salvelinus namaycush*) are a salmonid fish species endemic to North America with substantial cultural, ecological, and economic importance. Across their range, lake trout are often the keystone predator of lentic ecosystems (Ryder *et al.* 1981) and historically supported valuable commercial and subsistence fisheries (Waters 1987; Hansen 1999; Brenden *et al.* 2013). Lake trout express a large degree of sympatric phenotypic variation (Muir *et al.* 2016) making them a useful species for exploring the genomic basis for phenotypic diversity. Multiple morphotypes exist across the species range (Muir *et al.* 2016; Marin *et al.* 2017), with diversification largely associated with the ability to exploit resources and habitats at varying depths in large post-glacial lakes (Zimmerman *et al.* 2006; Stafford *et al.* 2013; Muir *et al.* 2014; Marin *et al.* 2017). In the Great Lakes, trophic specialization has resulted in the evolution of three widely recognized morphotypes — leans, siscowets, and humpers — that are differentiated by patterns of skin pigmentation, size-at-age, body shape, tissue lipid content, habitat use, and diet (Thurston 1962; Eschmeyer and Phillips 1965; Burnham-Curtis 1994; Harvey *et al.* 2003; Alfonso 2004; Zimmerman *et al.* 2007; Zimmerman *et al.* 2009; Goetz *et al.* 2013). Similar patterns of divergence exist in other lake trout populations (Blackie *et al.* 2003; Zimmerman *et al.* 2006; Hansen *et al.* 2012; Marin *et al.* 2016; Chavarie *et al.* 2015), with some degree of morphological and phenological variation existing among individuals of the same morphotype (Bronte 1993; Bronte and Moore 2007).

Previous studies have evaluated differences in gene expression and signals of adaptive divergence between lake trout morphotypes (Goetz *et al.* 2010; Bernatchez *et al.* 2016; Perreault-Payette *et al.* 2017). However, no study has explicitly evaluated which loci control variation in specific traits that underly morphotype divergence. Additionally, these studies have relied on *de novo* assembled markers distributed anonymously across the genome. Although these approaches can be powerful (Davey *et al.* 2013), fully interpreting results requires some knowledge of how loci are ordered along chromosomes. All scans for adaptively significant loci and genotype-phenotype associations inherently take advantage of linkage disequilibrium between genotyped markers and causal loci. Without knowing the relative locations of loci, it can be difficult to determine if genotype-phenotype associations or signals of selection are associated with a single genomic region or multiple regions distributed widely across the genome. Information on the order of loci along chromosomes can be readily attained via linkage mapping or assembly of a reference genome; however, linkage maps are often needed *a priori* to produce chromosome-scale genome assemblies.

Linkage maps have been used to map loci associated with disease resistance (Houston *et al.* 2008; Moen *et al.* 2009), life history and physiological trait variation (Rogers and Bernatchez 2007; Miller *et al.* 2012; Gagnaire *et al.* 2013a; Sutherland *et al.* 2017; Pearse *et al.* 2019), and commercially valuable traits (Gonzalez-Pena *et al.* 2016) in salmonids and have been instrumental in the assembly of salmonid reference genomes (Lien *et al.* 2016, Christensen *et al.* 2018a, 2018b; Pearse *et al.* 2019; Sävilammi *et al.* 2019). A linkage map for lake trout would enable the application of cutting-edge genomic tools to questions in lake trout management and evolution and would aid in the identification of loci underlying phenotypic variation and local adaptation. Specifically, a linkage map would increase the strength of inference from genome-wide association studies and scans for selection (Bradbury *et al.* 2013; Gagnaire *et al.* 2013b; McKinney *et al.* 2016) and allow for the localization of quantitative trait loci (Peichel *et al.* 2001; Qiu *et al.* 2018) and tracts of admixture and homozygosity, and the estimation of historical effective population sizes and admixture dynamics (Hollenbeck *et al.* 2016; Leitwein *et al.* 2018). This information would be valuable for selecting stocks for reintroduction and translocation and for estimating the adaptive potential of intact populations under changing climate and abiotic conditions (Leitwein *et al.* 2016; Bay *et al.* 2017).

Comparative analysis of linkage maps and genome assemblies from related species can also shed light on chromosomal evolution and speciation (Rastas *et al.* 2015; Sutherland *et al.* 2016; Hale *et al.* 2017). Chromosomal inversions appear to have played an important role in speciation and adaptive divergence within the salmonid lineage (Miller *et al.* 2012; Sutherland *et al.* 2016, Pearse *et al.* 2019) and within other taxa (Lowry and Willis 2010; Küpper *et al.* 2016; for review see Wellenreuther and Bernatchez 2018). Instances of reduced hybrid fitness and hybrid inviability are widespread within the family Salmonidae (Leary *et al.*, 1993; Fugjiwara *et al.* 1997; Muhlfeld *et al.* 2009). Information on the locations of inversions differentiating species and phenotypically divergent populations could shed light on the genetic basis for these phenomena. Inversions can contribute to isolation between species and populations because they can suppress recombination over large chromosomal regions, allowing for adaptive differences to accumulate between inverted and non-inverted haplotypes even in the presence of gene flow (Berg *et al.* 2017; Wellenreuther and Bernatchez 2018). Inversions can also produce post-zygotic isolation between incipient species if crossing over within heterozygous individuals results in formation of abnormal or inviable gametes (Wellenreuther and Bernatchez 2018). An improved understanding of the extent to which pericentric (including the centromere) and paracentric (outside the centromere) inversions can accumulate between salmonid species over varied evolutionary time scales, could provide clues about pre- and post-zygotic isolation mechanisms that contributed to adaptive divergence and incipient speciation within salmonids.

Linkage maps have been constructed for multiple salmonid species including rainbow trout (Miller *et al.* 2012; Palti *et al.* 2012; Gonzalez-Pena *et al.* 2016), chinook salmon (Brieuc *et al.* 2014; McKinney *et al.* 2016; McKinney *et al.* 2019), coho salmon (Kodama *et al.* 2014), sockeye salmon (Everett, Miller and Seeb 2012; Larson *et al.* 2015; Limborg *et al.* 2015), chum salmon (Waples *et al.* 2016); pink salmon (Spruell *et al.* 1999; Lindner *et al.* 2000), Atlantic salmon (Moen *et al.* 2008; Lien *et al.* 2011; Brenna-Hansen *et al.* 2012; Gonen *et al.* 2014), Arctic char (Nugent *et al.* 2017; Christensen *et al.* 2018a), brook trout (Sauvage *et al.* 2012; Sutherland *et al.* 2016; Hale *et al.* 2017), brown trout (Leitwein *et al.* 2017), European grayling (Sävilammi *et al.* 2019), lake whitefish (Rogers and Bernatchez 2007; Gagnaire *et al.* 2013a), and European whitefish (De-Kayne and Feulner 2018). No linkage map has been constructed for lake trout (but see May *et al.* 1979, Johnson *et al.* 1987, for work on segregation patterns in lake trout x brook trout hybrids), although the lake trout karyotype has been characterized in multiple previous studies (Phillips and Zajicek 1982; Reed and Phillips 1995) providing a reference for the number of expected chromosomes.

Here we present a high-density linkage map for lake trout generated using restriction site associated DNA (RAD) capture (Rapture; Ali *et al.* 2016), a modified RAD sequencing protocol that allows variable loci to be preferentially genotyped. The map was used to characterize the lake trout karyotype, estimate recombination rates, determine centromere locations, map the sex determination locus, and identify chromosomal inversions and translocations differentiating lake trout from other salmonids. We demonstrate the utility of the linkage map by using available phenotype data to map quantitative trait loci (QTL) associated with pigmentation patterns, growth and condition related traits, and variation in body shape — all traits hypothesized to be adaptive in lake trout and other salmonids.

## Materials And Methods

### Linkage mapping families

**Figure 1:**
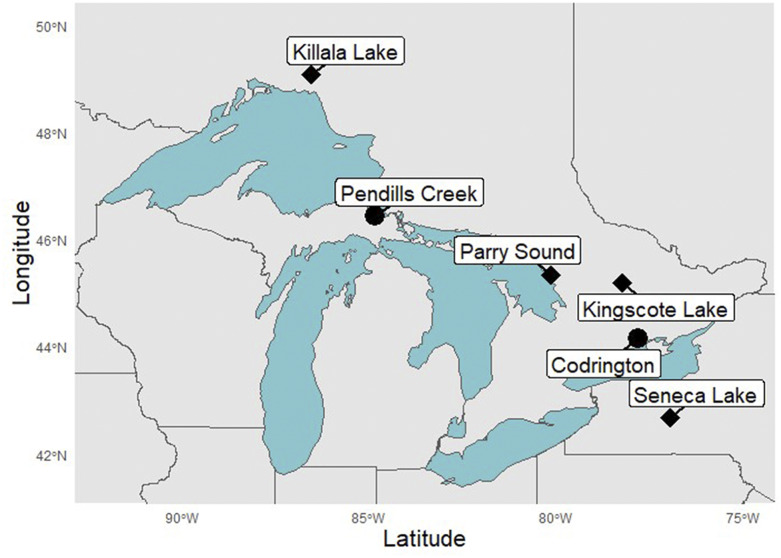
Map displaying the locations of hatchery facilities (dots) and locations of wild progenitor populations (diamonds) used for mapping. Locations of hatchery facilities used for conducting crosses are marked with black circles. The locations of the progenitor populations are identified with black diamonds. Longitude is displayed on the Y-axis and latitude is displayed on the X-axis.

Two F1 full-sibling families were created by crossing Seneca Lake hatchery strain females with Parry Sound strain males (Table 1, Figure 1). The Seneca Lake strain was founded using individuals from Seneca Lake, New York and this strain has contributed disproportionately to restoring lake trout populations in the Great Lakes (Scribner *et al.* 2018). The Parry Sound strain was founded by wild individuals collected from Georgian Bay in Lake Huron. The Seneca and Parry Sound strains are genetically divergent (F_ST_ = 0.089) based on a previous study using microsatellites (Scribner *et al.* 2018). Crosses were produced in 2017 using adult lake trout and housed at Pendills Creek National Fish Hatchery (U.S. Fish and Wildlife Service, Figure 1). Eggs were fertilized, incubated in Heath trays at ambient temperature, and raised until swim-up phase. Offspring were then killed using a lethal dose of MS-222 and preserved in 95% ethanol. Genetic sex was determined for offspring using a sdY presence-absence quantitative PCR (qPCR) assay designed using the approach of Anglès d’Auriac *et al.* (2014; see Trait Mapping methods below). These families were ultimately used for constructing the linkage map and localizing the lake trout sex determination locus.

**Table 1:** Family IDs, cross type (diploid or gynogenetic diploid), number of genotyped offspring per family, and maternal and paternal origins for the five families used for linkage and QTL mapping.

Family	Type	No. Offspring	Mother Origin	Father Origin
S1	Diploid	88	Seneca Lake	Parry Sound
S2	Diploid	91	Seneca Lake	Parry Sound
P1	Diploid	91	Killala X Kingscote F1	Killala X Kingscote F1
P3	Diploid	88	Killala X Kingscote F1	Killala X Kingscote F1
G1	Gynogenetic Diploid	45	Killala X Kingscote F1	None

An additional F2 half-sibling family was created using adult lake trout from the Killala Lake hatchery strain and wild individuals from Kingscote Lake, Ontario (Table 1, Figure 1). The Killala Lake strain was founded by individuals from Killala Lake, Ontario, which is within the Lake Superior drainage. This hatchery strain is most similar to lean form hatchery strains derived from Lake Superior based on a previous allozyme genotyping study (Marsden *et al.* 1993). Individuals from the Kingscote Lake strain also resemble lean lake trout; however, they are small bodied and lack spots and vermiculations (Wilson and Evans 2010). Examination of F2 offspring at age 3 revealed substantial variation in pigmentation, weight and length at age, and body shape among individuals. These traits are commonly recognized as being adaptively differentiated between lake trout populations and ecophenotypes (Eshenroder 2008; Muir *et al.* 2016). Body shape and early growth rate in particular have been recognized as important traits for differentiating lean, siscowet, and humper ecophenotypes (Moore and Bronte 2001; Hansen *et al.* 2016). The observation that skin pigmentation patterns vary between ecophenotypes and across depth strata in some lake trout populations also suggests that pigmentation traits might be an important axis of ecophenotypic divergence within lake trout (Zimmerman *et al.* 2006). The F2 Kingscote x Killala family was used for linkage map construction, localization of the sex determination locus, and QTL mapping. Crosses, culture conditions, and phenotyping procedures are described below.

Initial Kingscote x Killala F1 crosses were produced using adult lake trout using a 2x2 factorial mating design. In 2012, mature adults from initial crosses were mated to produce F2 families. Eggs from each family were incubated in Heath trays at ambient temperature (2-5°). Prior to swim-up, hatched sac fry from families were transferred to 36L laundry tubs (200 fry per tub) where they remained until age 1+. Families were manually fed 1% of tank biomass twice-daily and family sizes were periodically reduced by culling to avoid overcrowding. At age 1+, families were transferred to 700L circular tanks with ambient lighting and fed to satiation on an EWOS pellet diet. At age 3, fork length and weight were determined and lateral photographs were collected using the protocol from Bernatchez *et al.* (2016). Fish were photographed using a Nikon Coolpix P7700 digital camera with a focal length of 50mm mounted on a tripod in fixed position. Fish were photographed with the head facing to the left and were cradled in a stretched mesh net as in Zimmerman *et al.* (2006) in order to avoid distorting body shape. Fin clips were collected and preserved in 95% ethanol. Photographs were later used for morphometric analysis and scoring individuals for presence-absence of spots and vermiculations (see Trait Mapping methods section below).

An additional gynogenetic diploid family was created using a female F1 resulting from initial Kingscote x Killala crosses using a protocol similar to that of Thorgaard *et al.* (1983). This family was used for mapping centromeres using half-tetrad analysis (Thorgaard *et al.* 1983; Limborg *et al.* 2016). Sperm from a male lake trout was diluted 10:1 using sperm extender (9.2 g Tris buffer, 1.05 g citric acid, 4.81 g glycine, 2.98 g KCl, 100g PVP-40, and 1 liter of distilled water), mixed thoroughly in a 9x13x2 inch glass pyrex dish, placed on ice, and irradiated for 2 min using a 25-watt germicidal UV lamp placed 20 centimeters from the dish. Eggs and sperm were then mixed and sperm was activated by adding water. Ten minutes after fertilization, eggs were heat shocked at 26° for 10 min, water hardened, transferred to Heath trays for incubation, and raised using the same conditions described for diploid families. All Kingscote X Killala families were produced at the Codrington Fisheries Research Facility (Ontario Ministry of Natural Resources and Forestry; Figure 1; Codrington, Ontario). This facility has a surface water supply which undergoes seasonal and diel temperature variation ranging between 2-5° in winter and 9-16° in summer.

### Sample preparation

For all Kingscote x Killala families, DNA from offspring and parents (Table 1) was extracted using the high-throughput SPRI bead-based extraction protocol described in Ali *et al.* (2016) with Serapure beads (described in Rohland and Reich 2012) substituted for Ampure XP beads. For the Seneca x Parry Sound crosses, DNA was extracted using Qiagen DNeasy Blood and Tissue extraction kits (69506, Qiagen, Hilden, Germany) using manufacturer recommendations. DNA quality was initially assessed using a Nanodrop 2000 Spectrophotometer (Thermo Fisher Scientific, Waltham, Massachusetts) by evaluating 260/230 and 260/280 absorbance ratios. Samples were diluted to less than 100ng/ul based on Nanodrop readings, then diluted 10-fold before determining double-stranded DNA (dsDNA) concentrations using Quantit Picogreen assays (Thermo Fisher Scientific, Waltham, Massachusetts).

### Sequencing library preparation

DsDNA concentrations were normalized to 10ng/ul using an Eppendorf epMotion 2750 TMX liquid handling robot (Eppendorf, Hamburg, Germany) before proceeding with the bestRAD protocol and RAD-capture using 100ng of total input DNA (Ali *et al.* 2016). Modifications to the protocol are noted below with a detailed description of methods provided in supplementary material (Document S1). First, the enzyme PstI was substituted for SbfI and PstI was heat-killed at 80° rather than 65°. After ligating bestRAD adapters and pooling samples, shearing was carried out using a Covaris E220 Ultrasonicator (Covaris Inc., Woburn, Massachusetts) using the recommended settings for a 300bp mean fragment length. Finished libraries were amplified for 10 cycles, pooled equally in sets of two, and bead cleaned twice using a 0.9:1 bead-to-DNA ratio Ampure XP cleanup (A63881, Beckman Coulter, Brea, California). The two resulting pools were then enriched for 58,889 RAD loci that were previously found to be variable in lake trout populations in the Great Lakes, Seneca Lake, Ontario, Montana, and Alaska using the RAD-capture protocol (Ali *et al.* 2016). Target enrichment reactions were carried out using a MyBaits Custom Target Enrichment kit using manufacturer recommendations (MycroArray, Ann Arbor, Michigan; Protocol Version 3; for more information on capture and bait selection see Document S1). Finished capture reactions were amplified for an additional 9 cycles, pooled, and sequenced in three lanes of an Illumina HiSeq X instrument (2 X 150 bp paired end reads; Illumina, San Diego, California) by the Novogene Corporation (Novogene, Sacremento California).

### Bioinformatics and genotyping

Read quality was initially assessed using FastQC v0.11.5 (Andrews 2014), and a custom script was used to re-orient paired end reads such that individual specific barcodes and restriction enzyme overhang sequences were always located at the beginning of the first read. Reads were demultiplexed using process_radtags v2.2, duplicate reads were removed using clone_filter v2.2 (Catchen *et al.* 2013; Rochette *et al.* 2019), and adapter sequences were clipped from reads using Trimmomatic v0.36 (Bolger *et al.* 2014). At this point, we produced two sets of fastq files: one conservatively filtered dataset used for *de novo* assembly of RAD loci and a slightly less conservatively filtered dataset used for calculating genotype likelihoods that would ultimately be used for linkage mapping and other analyses. For the *de novo* assembly dataset, reads were trimmed whenever the mean base quality across a sliding window of 4bp dropped below Q20, read pairs were removed if one or both reads in a pair were less than 140bp in length after trimming, and reads were cropped to a length of 140bp such that all reads were of identical length. For the dataset used to calculate genotype likelihoods, reads were trimmed whenever the mean base quality across a sliding window of 4bp dropped below Q15 and excluded if one or both reads in the pair were less than 50bp after trimming.

The stringently filtered dataset (read length =140bp, trimming threshold of Q20) was used to assemble RAD loci *de novo* using modules available in Stacks v2.2 (Rochette *et al.* 2019). RAD loci were identified for individuals using ustacks v2.2, which was run with a minimum depth of coverage of 3 (-m 3), a maximum distance between stacks of 3 (-M 3), a maximum distance to align secondary reads to primary stacks of 2 (-N 2), a minimum of 2 stacks at each *de novo* locus (–max_locus_stacks 2), and disabling calling haplotypes from secondary reads (-H). We then created a catalog of RAD loci for the parents of crosses using cstacks v2.2, allowing for up to two mismatches between sample loci when building the locus catalog (-n 2). Putative RAD loci alleles for all individuals were matched to this catalog using sstacks v2.2, converted to bam format using tsv2bam v2.2, and then assembled using gstacks v2.2. Consensus sequences for RAD loci were obtained by passing the “–fasta-loci” flag to the populations v2.2 module. The fasta file containing RAD locus consensus sequences was normalized using Picard NormalizeFasta v2.8 (http://broadinstitute.github.io/picard/), indexed using bwa index v7.15 (Li 2013) and samtools faidx v1.3 (Li *et al.* 2009), and used as a *de novo* reference for subsequent analysis.

Next, the larger set of variable length paired end reads that were trimmed using a Q-threshold of 15 were mapped to the *de novo* assembly using bwa mem v7.15 (Li 2013) with default setting. Genotype likelihoods were calculated for single nucleotide polymorphisms (SNPs) within RAD loci using Lepmap3 v0.2 and associated modules (Rastas *et al.* 2015). SAM files produced by bwa-mem were converted to bam format and sorted using samtools v1.3, then converted to mpileup format using a minimum mapping quality of 30 and a minimum base quality of 20. The resulting file was filtered using the script pileupParser2.awk using a minimum read depth of 3 and a missingness threshold of 0.3. Genotype likelihoods were calculated using the pileup2posterior.awk script distributed with LepMap3 v0.2 (Rastas *et al.* 2015, Rastas 2017). We opted to use pileup2posterior.awk to calculate genotype likelihoods because the LepMap3 pipeline was originally validated using likelihoods calculated using this program (Rastas 2017).

### Linkage map construction

Linkage mapping and additional data filtering were carried out using various programs distributed with LepMap3 v0.2 (Rastas 2017). First, any missing parental SNP genotypes were imputed using ParentCall2. Second, SNPs showing evidence of segregation distortion were removed using Filtering2 with a p-value (–dataTolerance) threshold of 0.01. We required that SNPs be informative for linkage mapping in at least 1 family and removed SNPs with minor allele frequencies less than 0.05.

SNPs were assigned to linkage groups (LGs) using SeparateChromosomes2 run with logarithm of odds ratios (LOD) thresholds ranging from 8 to 60 and a minimum LG size of 50 SNPs. No single LOD threshold produced the expected number of LGs (n = 42; Phillips and Zajicek 1982; Reed and Phillips 1995). Beginning with the map produced using a universal LOD threshold of 10, we determined the LOD thresholds needed to further split each LG by running SeparateChromosomes2 using all LOD thresholds between 10 and 60 and specifying the LG targeted for additional splitting using the “lg” and “map” flags (similar to Christensen *et al.* 2018a).

We determined that the largest 8 of the initial 30 LGs could be split using LOD thresholds ranging from 11-52, with the remaining 22 LGs remaining intact for all LOD thresholds between 10 and 60. The 8 largest LGs were split using the maximum LOD threshold that resulted in a new LG containing more than 50 SNPs, resulting in 42 LGs. Unassigned singleton SNPs were then joined to this map using JoinSingles2All run iteratively with a LOD threshold of 10 and a minimum LOD difference of 5.

The order of SNPs was initially determined by running 20 iterations of OrderMarkers2 and selecting the order with the highest likelihood for each LG. LGs were further refined by evaluating LOD matrices (output using computeLODscores = 1). For each SNP, the vector of LOD scores corresponding to possible map positions was normalized such that values ranged from 0 to 1. SNPs were removed if the maximum LOD score was less than 1 standard deviation from the mean or if more than one LOD ‘peak’ was observed for any given SNP, indicating the existence of multiple mapping positions of similar likelihood. LOD peaks were identified using the findPeaks function from the R package pracma v2.2.5, a minimum normalized peak height of 0.95 and a minimum distance between peaks greater than 25% of the length of the vector of mapping positions. RAD loci were removed from the data set if associated SNPs mapped to more than one LG. Finally, the dataset was thinned to include a single SNP for each RAD locus, with preference given to the SNP closest to the PstI restriction cut site. We opted to thin SNPs after determining which loci could be effectively mapped in order to maximize the number of unique RAD loci on the map. Maps for each LG were then reconstructed using the evaluateOrder and improveOrder = 1 options from OrderMarkers2, with SNPs that failed the above filtering criteria flagged for removal using the removeMarkers option.

Finally, LGs were inspected for possible mis-ordering using LMPlot and any LG marked with possible errors were reordered using OrderMarkers2 for an additional 60 iterations. The linkage map was further improved by trimming SNPs from the ends of LGs based on manual inspection of LOD matrix plots and alignment to rainbow trout, Arctic char, and Atlantic salmon (*Salmo salar*) genome sequences (see Homology section below). An additional 10 iterations of ordering were conducted after removing potential erroneously placed SNPs from the ends of LGs. Final LGs were sorted based on their number of mapped SNPs and named as Sna1-Sna42. Both male and female linkage maps were output by the program.

### Centromere mapping

We identified centromeres by estimating the frequency of second division segregation (*γ*) across linkage groups using half-tetrad analysis conducted on gynogenetic diploid offspring from family G1 (Thorgaard *et al.* 1983). Cells of gynogenetic diploid offspring contain two of the four possible meiosis II products (a half-tetrad) and the frequency of heterozygous offspring can be used to estimate the frequency of recombination events between the locus in question and the centromere (Thorgaard *et al.* 1983). Reads for these individuals were aligned to *de novo* assembled RAD loci, sorted and indexed using samtools v1.3 (Li *et al.* 2009), and variable positions within RAD loci were genotyped using freebayes v1.1.0 (Garrison and Marth 2012). Genotypes were called without applying population or binomial observation priors, an assumed contamination probability of 1%, a minimum base quality of 20, and a minimum mapping quality of 20. Called loci were then converted to their simplest representation using vcfallelicprimatives (https://github.com/vcflib/vcflib; vcflib v1.0.0) and loci with more than 2 alleles and indels were removed, such that only SNPs remained. Genotypes were set to missing if there was less than 1 order of magnitude difference in genotype likelihoods between the called genotype and the second most likely genotype using vcftools v0.1.16 (GQ >10; Danecek *et al.* 2011). SNPs were removed from the dataset if more than 30% of individuals were missing genotypes or if the frequency of the minor allele was less than 0.05. SNPs were further excluded if they were not placed on the linkage map, not called heterozygous in the mother, or if both possible homozygous genotypes were not observed in offspring. The mother was removed from the dataset at this point, and observed heterozygosity for the offspring (*y*) was calculated using the hwe function from SeqVarTools v1.20.2 (Gogarten *et al.* 2017; https://github.com/smgogarten/SeqVarTools). Centromeric regions were delineated as the region between the first and last markers with y-values less than 0.1 (as in Limborg *et al.* 2016).

Results were cross-validated and improved upon using the RFm method (Limborg *et al.* 2016) applied to the phased genotypes of progeny from families S1, S2, P1, and P3. Counts of maternal recombination events were reported using OrderMarkers2 with outputPhasedData = 1 and used to calculate RFm across all maternal haplotypes and identify putative centromeric regions using a cut-off value of 0.45 as suggested in Limborg *et al.* (2016). The correct centromeric locations for acrocentric and telocentric chromosomes were identified by selecting the region containing, or neighboring, the lowest y-values from half-tetrad analysis.

### Homology

RAD loci were aligned to the reference genomes for Arctic char (RefSeq Accession: GCF_002910315.2), Rainbow trout (*Oncorhynchus mykiss*; RefSeq Accession: GCF_002163495.1) and Atlantic salmon (RefSeq Accession: GCF_000233375.1) using bwa mem v7.15 (Li 2013). RAD loci were assigned to their respective linkage map positions, and male and female linkage maps were visualized relative to their order along homologous chromosomes using ggplot2 v3.2.1 (Wickham and Chang 2008). Chromosomes were considered homologous if 50 or more mapped RAD loci aligned to a chromosome with mapping qualities greater than MQ60. The map was also compared with a linkage map for brook trout (*Salvelinus fontinalis*; Sutherland *et al.* 2016) using the program MapComp (Sutherland *et al.* 2016; https://github.com/enormandeau/mapcomp) and the Arctic char genome as an intermediate reference in order to detect large structural variants differentiating the two species.

Putative chromosomal inversions were detected by manually inspecting plots produced by mapping the lake trout linkage map to divergent references. Inversion breakpoints were defined by the coordinates with the greatest discrepancy between the divergent physical map and the female linkage map we constructed. Inversions were classified as pericentric if putative inversion coordinates overlapped centromere mapping positions. Inversions differentiating lake trout and brook trout were detected by manually inspecting dot plots produced by MapComp.

### Trait mapping

Offspring from diploid Kingscote x Killala crosses were phenotyped for fork length (FL), weight (WT), and condition factor (CF) at age 3. Additionally, photographs collected at age 3 were used to score individuals for presence-absence of spots and vermiculations (VPA) and two composite variables (PCA1 and PCA2) summarizing variation in body shape. Body shape variables were derived by performing a principal-components analysis (PCA) on the coordinates of morphometric landmarks that were normalized for slight differences in fish position and rotation using generalized Procrustes analysis. Using available photographs from families P1 and P3, we placed landmarks using tpsDIG v2 (Rohlf 2005) consistent with those described in Muir *et al.* (2014). Landmark coordinates were normalized and rotated using generalized Procrustes analysis conducted using the function gpagen from the R-package geomorph v3.1.1 (Adams and Otárola‐Castillo 2013). Four of 20 landmarks could not be consistently placed using available images (1,6,7,10) and were therefore excluded from the analysis. Synthetic variables PCA1 and PCA2 were calculated by performing PCA on the resulting normalized coordinates and extracting scores for the first two axes. PCA was carried out using the function prcomp from the R-package stats v3.5.3. VPA, PCA1, and PCA2 phenotypes were available for 143 of 179 individuals. Fork length, condition factor, and weight phenotypes were collected for 179 of 179 individuals.

Phased SNP genotypes for offspring were extracted from the final map files reported by OrderMarkers2 using the script map2genotypes.awk from LepMap3 v0.2. QTL mapping was then carried out for traits of interest using the R-package qtl2 v0.2 and associated functions (Broman *et al.* 2019). All traits were mapped to sex-averaged linkage map coordinates. Prior to QTL mapping, pseudo-markers were added to the map using insert_pseudomarkers with a step size of 1cM and genotype probabilities were calculated using calc_genoprob. A kinship matrix was calculated using the calc_kinship function using genotype probabilities. A thinned subset of markers obtained using calc_grid (step = 3) and probs_to_grid was used as input for the calc_kinship function. QTL scans were carried out using scan1 and suggestive QTL peaks were identified using find_peaks (drop = 2, peakdrop = 2, threshold = 3). Traits with approximately normal distributions (FL, CF, WT, PCA1, PCA2) were mapped using a mixed linear model with the kinship matrix included as a random effect (model = “normal” and kinship options in qtl2). Presence-absence of vermiculations and spots (VPA) was mapped as a binary trait (model = “binary” in qtl2). The kinship matrix was not included as a random effect in the binary trait mapping model because this option was not available in qtl2. For each identified LOD peak, 95% credible intervals were calculated using the function find_peaks (prob = 0.95, peakdrop = 2, threshold = 3). Finally, p-values were calculated by comparing observed LOD scores for each peak with a null distribution obtained from permuting the data 1000 times. Permutations were carried out using the function scan1perm using the same settings as the original tests and p-values were calculated using the ecdf function. The proportion of phenotypic variation explained (PVE) by each QTL peak was calculated from LOD scores and sample sizes using the equation $PVE=1-10^{-\left(\frac{2}{\text{n}}\right)\text{*LOD}}$ (Broman and Sen 2009). Candidate genes for significant LOD peaks (*P* < = 0.05) were identified by mapping RAD loci within 95% credible intervals to the Arctic char genome and determining the three genes closest to each mapping position using the program bedtools closest v2.26 (Quinlan and Hall 2010). Genes were considered candidates if they were within 50Kb of the mapping position of a RAD locus falling within the identified QTL mapping interval.

We also mapped the sex determination region using the binary trait model using qtl2 and assessed significance using the same methodology described above. The sexually dimorphic on the Y chromosome gene (*sdY*) is believed to underly sex determination in lake trout and some other salmonids (Yano *et al.* 2013). We designed a *sdY* presence-absence melt curve qPCR assay (similar to Anglès d’Auriac *et al.* 2014) using the lake trout *sdY* and *18S* primers described in Yano *et al.* (2013). *18S* served as an internal amplification control. Each reaction was carried out using a 0.4 uM concentration of primers sdYE2S1 (CCCAGCACTGTTTTCTTGTCTCA) and sdYE2AS1 (TGCTCTCTGTTGAAGAGCATCAC), and a 0.04uM concentration of primers 18SS (GTYCGAAGACGATCAGATACCGT) and 18SAS (CCGCATAACTAGTTAGCATGCCG). Reaction volumes were 20uL and contained 10ul of Forget-Me-Not EvaGreen qPCR mastermix (31045, Biotium, Fremont, California), 2.5 uL of template DNA, and 7.5uL of primers eluted in water. *18S* and *sdY* were amplified in a two-step multiplex reaction using a 2-minute heat activation step at 95° followed by 40 cycles of denaturation at 95C for 5 sec and annealing/extension at 60° for 30 sec. Melt curve analysis was carried out on PCR product for temperatures between 60° and 95° using 0.1° temperature shifts and a 3 sec pause between temperature shifts. We first tested the assay on a subset of 32 individuals of known sex (16 males and 16 females), including the parents used for crosses, in order to verify that males and females could consistently be differentiated based on the presence of a male specific *sdY* peak in the derivative of the melt curve. Offspring from all diploid families were subsequently genotyped using the same reaction conditions described above. At least one known male and one known female were included on each plate as a control. Sex locus mapping was carried out with sdY presence being coded as 1 and sdY absence coded as 0.

### Recombination rate estimation

We estimated sex averaged recombination rates for each chromosome by performing a simple linear regression of pairwise physical distance (base pairs) against genetic distance (cMs) and requiring the intercept to pass through 0. In order to evaluate a pair of RAD loci, we required that they map to the same chromosome on the Arctic char, rainbow trout, or Atlantic salmon genome assemblies and only retained scaffolds and chromosomes with greater than 50 mapped RAD loci for which the mapping quality was 60. For each LG, 100 pairs of RAD loci mapping to the same chromosome were randomly sampled from all possible pairs and recombination rate (cM/MB) was estimated using the slope of the resulting regression. This process was repeated 100 times using alignments against the Arctic char, rainbow trout, and Atlantic salmon genomes. The mean of the distribution of estimates was reported as the chromosome specific recombination rate, and separate values were reported for alignments against the three different divergent reference genomes. Regressions were carried out using the R-package lm and recombination rate estimates were visualized using ggplot2 v3.2.1 (Wickham and Chang 2008). This process was repeated for male and female maps in order to obtain sex specific chromosomal recombination rates.

### Data availability

Sequencing data for all individuals used for linkage and QTL mapping has been made publicly available in a NCBI sequence read archive (PRJNA608030). Map information, phenotypes, and genotypes used for QTL and sex locus mapping are available in supplementary material (Document S2). Supplemental material available at figshare: https://doi.org/10.25387/g3.11908326.

## Results

### Bioinformatics and genotyping

We obtained a mean of 2,685,178 demultiplexed paired end (PE) reads for offspring from diploid crosses (range = 660,474 – 4,317,086, SD = 598,973.3) and a mean of 4,701,286 reads for parents (range = 3,483,973 – 5,868,449, SD = 787,251.1). On average, 21.97% of reads were removed by clone_filter for these individuals. *De novo* assembly of RAD loci with gstacks produced 146,525 RAD loci ranging in size from 140 to 754 bp in length. Between 92.86% and 95.20% of reads were mapped to *de novo* assembled RAD loci (mean = 93.52%, SD = 0.33%) using bwa mem. The Lepmap3 genotyping pipeline reported genotype probabilities for 212,158 SNPs, 147,920 of which were informative for linkage mapping. Of those, 72,549 SNPs passed missingness, segregation distortion, and minor allele frequency filters.

For gynogenetic diploid offspring, we obtained an average of 3,873,649 PE reads (range = 1,517,646 – 5,789,490, SD = 1,004, 905). We generated 3,536,915 reads for the mother of this family. On average, 32.63% of reads for these samples were removed by clone_filter. Between 89.0% and 89.6% of those reads were mapped to the *de novo* assembly using bwa mem (mean = 89.3%, SD= 0.12%). After genotyping with freebayes and filtering data to remove non-informative markers, we identified 893 SNPs that were informative for half-tetrad analysis.

### Linkage and centromere mapping

**Figure 2:**
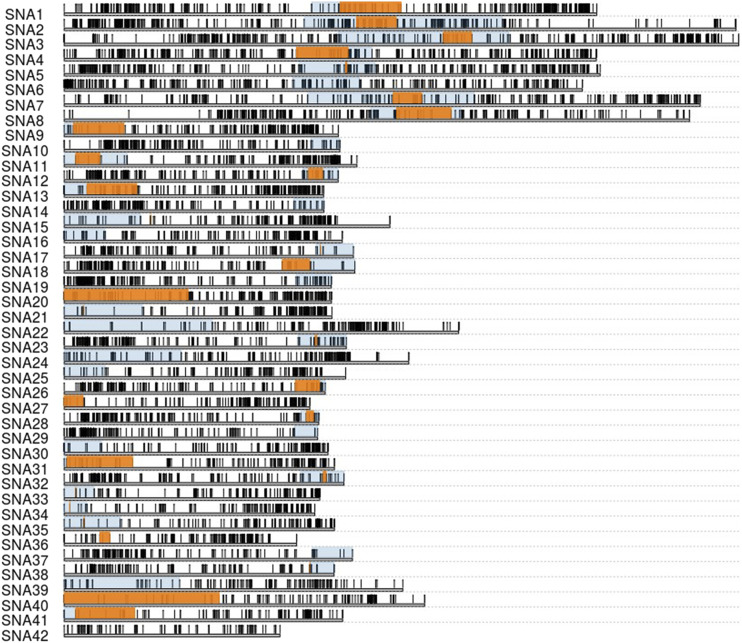
Map Locations of 15,740 RAD loci along 42 lake trout linkage groups. Orange boxes highlight centromeres identified using half tetrad analysis with a y-threshold of 0.1. Blue boxes span the intervals of centromeres identified using the RFm method (Limborg *et al.* 2016) combined with half-tetrad analysis. Locations are in centimorgans on the female linkage map.

We were able to assign 15,740 RAD loci to LGs with between 878 and 113 loci mapped to each LG (Figure 2, Table 2, Table S1). The total male map length was 2043.41cM and the female map was 2842.22 cM (overall female:male map ratio = 1.391). Male LG map lengths ranged from 1.97 cM -92.87 cM, while female LG map lengths ranged from 43.07 cM – 134.64 cM (Table 2). SNPs were mapped to between 60 and 244 unique positions on linkage groups. As expected, we identified 42 LGs, 8 of which were metacentric and 34 that were acrocentric or telocentric (Table S1, Table S2). These linkage groups likely correspond to the 42 chromosomes identified by previous karyotyping studies (Phillips and Zajicek 1982; Reed and Phillips 1995). Half-tetrad analysis yielded centromere intervals for 7 of 8 metacentric chromosomes and 22 of 34 LGs identified as acrocentric or telocentric (Figure 2, Figure S8, Table S2). RFm analysis identified centromeres for 8 of 8 metacentric chromosomes and 32 of 34 acrocentric or telocentric chromosomes (Figure 2, Table S2). We were ultimately able to determine the location of centromeres for 41 out of 42 chromosomes using the two methods. We were not able to map the centromere for Sna42; however, this chromosome is likely acrocentric or telocentric based on the size of the linkage group relative to others (Table 2) and karyotyping work suggesting the existence of 34 acrocentric and telocentric chromosomes (Phillips and Zajicek 1982; Reed and Phillips 1995).

**Table 2:** Summary statistics for each of the 42 constructed linkage groups. No. Mapped Loci corresponds to the number of unique RAD contigs mapped to each linkage group. Male and Female map lengths are in centimorgans (cM). No. Unique Positions corresponds to the number of unique linkage map positions to which RAD loci were assigned. Female:Male Ratio is the ratio of Female Length and Male Length in centimorgans.

Name	No. Mapped Loci	Male Length (cM)	Female Length (cM)	Female:Male Ratio	No. Unique Positions
Sna1	878	85.28	106.3	1.246	217
Sna2	789	71.98	134.08	1.863	207
Sna3	761	59.12	134.64	2.277	244
Sna4	648	66.01	106.22	1.609	207
Sna5	618	50.95	106.98	2.100	221
Sna6	515	77.88	103.44	1.328	223
Sna7	514	73.49	126.94	1.727	195
Sna8	497	92.87	124.81	1.344	157
Sna9	460	48.39	54.72	1.131	112
Sna10	406	54.29	55.02	1.013	112
Sna11	404	42.38	58.43	1.379	100
Sna12	395	73.61	54.69	0.743	121
Sna13	389	34.02	51.78	1.522	116
Sna14	377	59.65	51.85	0.869	110
Sna15	360	48.12	65.02	1.351	101
Sna16	358	53.9	55.51	1.030	102
Sna17	357	48.72	57.73	1.185	95
Sna18	356	44.67	58.01	1.299	101
Sna19	348	41.15	53.32	1.296	109
Sna20	344	36.93	53.35	1.445	102
Sna21	340	41.96	53.44	1.274	92
Sna22	333	70.57	78.73	1.116	109
Sna23	332	61.14	56.31	0.921	106
Sna24	325	28.44	68.78	2.418	105
Sna25	322	63.3	56.18	0.888	98
Sna26	319	36.71	52.1	1.419	95
Sna27	317	33.52	49.03	1.463	94
Sna28	313	37.01	50.81	1.373	102
Sna29	312	48.84	50.59	1.036	86
Sna30	310	66.35	52.65	0.794	83
Sna31	307	36.9	53.94	1.462	93
Sna32	302	56.79	55.84	0.983	102
Sna33	286	50.85	51.02	1.003	89
Sna34	255	52.77	50.02	0.948	85
Sna35	244	42.17	53.94	1.279	94
Sna36	242	30.19	46.4	1.537	80
Sna37	225	26.84	57.54	2.144	82
Sna38	218	35.06	53.87	1.537	83
Sna39	194	22.56	67.59	2.996	91
Sna40	185	33.84	71.95	2.126	90
Sna41	172	2.12	55.58	26.217	60
Sna42	113	1.97	43.07	21.863	60

### Homology analysis

Alignment of the linkage map to divergent salmonid reference genomes revealed that the resulting map was highly congruent with existing assembled salmonid genomes (Figures S1-S6). Large synteny blocks were detected between lake trout linkage groups and the Arctic char genome for linkage groups Sna1-Sna41 (Table 3). Alignments suggested that Sna42 is syntenic with sal34; however, fewer than 50 loci with MQ60 mapped to this chromosome from Sna42. Syntenies were detected between lake trout and all rainbow trout and Atlantic salmon chromosomes. MapComp identified homologies with all brook trout linkage groups identified by Sutherland *et al.* (2016) (Figure S7).

**Table 3:** Synteny between lake trout linkage groups and Arctic char, rainbow trout, Atlantic salmon, and brook trout genomes. Arctic char, rainbow trout, and Atlantic salmon chromosomes were recorded if more than 50 RAD contigs from the lake trout linkage group were aligned to a chromosome with a mapping quality of 60. Brook trout linkage groups were recorded if more than 10 aligned markers were detected by MapComp. Graphical depictions of alignment location *vs.* linkage map position are available in Supplementary Figures S1-S7.

Lake Trout	Arctic Char	Rainbow Trout	Atlantic Salmon	Brook Trout
Sna1	Sal15	Omy6	Ssa24, Ssa26	BC6
Sna2	Sal1	Omy17	Ssa12	BC3
Sna3	Sal20	Omy12	Ssa03, Ssa13	BC8, BC14
Sna4	Sal18	Omy16, Omy23	Ssa01, Ssa19	BC1
Sna5	Sal6.1, Sal6.2	Omy2, Omy14	Ssa05	BC7
Sna6	Sal3	Omy21	Ssa07	BC2
Sna7	Sal27	Omy15, Omy18	Ssa16, Ssa29	BC5
Sna8	Sal13	Omy4, Omy10	Ssa04, Ssa23	BC4
Sna9	Sal26	Omy1	Ssa16	BC20
Sna10	Sal16	Omy5	Ssa10	BC17
Sna11	Sal32	Omy8	Ssa14	BC22
Sna12	Sal23	Omy10	Ssa04	BC9
Sna13	Sal2	Omy3	Ssa25	BC24
Sna14	Sal7	Omy9	Ssa15	BC30
Sna15	Sal9	Omy19	Ssa01	BC12
Sna16	Sal17	Omy16, Omy20	Ssa13, Ssa19	BC18
Sna17	Sal8	Omy25	Ssa09	BC33
Sna18	Sal33	Omy11	Ssa20	BC40
Sna19	Sal36	Omy22	Ssa21	BC26
Sna20	Sal11	Omy7	Ssa22	BC21
Sna21	Sal4q.1:29	Omy2	Ssa10	BC15
Sna22	Sal25	Omy1	Ssa18	BC36
Sna23	Sal22	Omy27	Ssa20	BC25
Sna24	Sal14	Omy4	Ssa06	BC31
Sna25	Sal19	Omy28	Ssa03	BC11
Sna26	Sal5	Omy29	Ssa11	BC10
Sna27	Sal31	Omy18	Ssa27	BC23
Sna28	Sal4q.2	Omy25	Ssa09	BC35
Sna29	Sal28	Omy8	Ssa15	BC19
Sna30	Sal10	Omy26	Ssa11	BC28
Sna31	Sal4q.1:29	Omy5	Ssa01	BC13
Sna32	Sal30	Omy14	Ssa14	BC34
Sna33	Sal14	Omy11	Ssa19	BC16
Sna34	Sal4p	Omy24	Ssa09	BC38
Sna35	Sal8	Omy20	Ssa28	BC27
Sna36	Sal37	Omy9	Ssa18	BC32
Sna37	Sal35	Omy3	Ssa02	BC29
Sna38	Sal24	Omy15	Ssa17	BC37
Sna39	Sal21	Omy13	Ssa02	BC42
Sna40	Sal12	Omy7	Ssa17	BC39
Sna41	Sal20	Omy13	Ssa06	BC14
Sna42	Sal34*^a^*	Omy19	Ssa08	BC41

a Sal34 appears to be homologous with Sna42, however fewer than 50 RAD contigs mapped to this chromosome.

**Figure 3:**
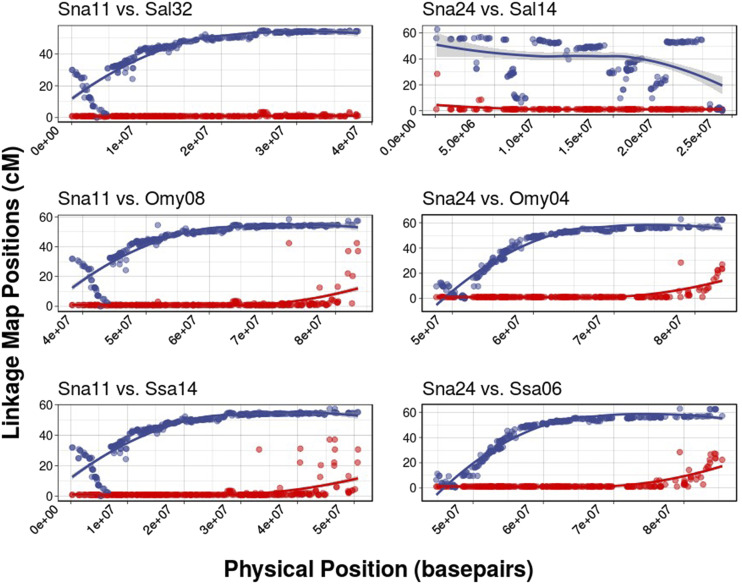
Examples of two linkage groups (Sna11 and Sna24) with evidence of inversions differentiating lake trout from other salmonids. Female lake trout linkage groups are colored blue (top curves). Male lake trout linkage groups are colored red (bottom curves). Sna11(first column) is differentiated from all homologs by a single large pericentric inversion spanning 0-30cM on the female linkage map (left side of each panel). Sna24 is differentiated from Omy04 and Ssa06 by an inversion spanning 0-10cM on the female map. It is unclear if the same inversion exists in Arctic char due to extensive structural differentiation relative to lake trout and other salmonids (Sna24 *vs.* Sal14).

The lake trout karyotype is differentiated from Arctic char by multiple Robertsonian translocations including one possible chromosomal fusion (Sal6.1 and Sal6.2) and four chromosomal fissions (Sal8, Sal14, Sal20, Sal4q.1.29). Sal6.1 and Sal6.2 are fused and Sal4q.1.29 is split into two LGs in lake trout, similar to the Arctic char linkage map presented by Nugent *et al.* (2017). The two *Salvelinus* species are also differentiated by at least 6 putative chromosomal inversions (Table 4), primarily on acrocentric or telocentric chromosomes. Arctic char chromosome Sal14 in particular appears to be the result of a fusion between Sna24 and Sna33. Sna24 also contains multiple chromosomal inversions that differentiate the two karyotypes (Figure 3). With the exception of inversions detected on Sna24, all putative inversions differentiating the two species were found to be near, or overlapping, the centromere (n = 5, Table 4). MapComp results suggest inversions on Sna10, Sna11, Sna24, and Sna34 are shared with brook trout; however, large inversions differentiating brook trout and lake trout were identified on Sna28 (brook trout BC35), Sna12 (brook trout BC9), and Sna23 (brook trout BC25).

**Table 4:** The first column is the lake trout linkage group in question and columns 2-4 list the approximate location of any detected inversions that differentiate species. The type of inversion is stated in parenthesis. Locations are listed in centimorgans on the female map. Whenever multiple inversions were detected on a chromosome, at least one was pericentric. Centromeres were not localized for Sna42, so centricity of inversions could not be determined.

Linkage Group	Arctic Char	Rainbow Trout	Atlantic Salmon
Sna1	—	—	—
Sna2	—	—	—
Sna3	—	—	—
Sna4	—	—	—
Sna5	—	—	—
Sna6	—	—	30-43 (Paracentric)
Sna7	—	—	—
Sna8	—	—	—
Sna9	—	—	—
Sna10	45-55 (Pericentric)	**	*
Sna11	0-30 (Pericentric)	0-30 (Pericentric)	0-30 (Pericentric)
Sna12	48-54 (Pericentric)	**	48-54 (Pericentric)
Sna13	—	—	—
Sna14	—	—	—
Sna15	—	—	—
Sna16	—	12-40 (Multiple Inversions)**	25-40 (Multiple Inversions)
Sna17	—	—	
Sna18	—	—	
Sna19	—	5-30 (Paracentric), 25 - 58 (Pericentric)	30-58 (Pericentric)
Sna20	—	0-10 (Pericentric)	0-10 (Pericentric)
Sna21	—	—	—
Sna22	—	—	—
Sna23	—	—	—
Sna24	0-57 (Multiple inversions)	0-12 (Pericentric)	0-12 (Pericentric)
Sna25	—	—	0-30 (Pericentric)
Sna26	—	—	—
Sna27	—	—	—
Sna28	—	—	43-52 (Pericentric)
Sna29	—	—	—
Sna30	*	0-10 (Pericentric)	—
Sna31	—	—	0-7 (Pericentric)
Sna32	—	—	—
Sna33	—	—	—
Sna34	0-30 (Pericentric)	*	*
Sna35	—	0-47 (Pericentric)	—
Sna36	—	—	—
Sna37	—	—	—
Sna38	—	—	—
Sna39	—	—	—
Sna40	—	—	—
Sna41	*	—	—
Sna42	—	35-43 (Unknown)	20-43 (Unknown)

* = suggestive evidence of structure variation but unable to determine if an inversion occurred.

** = Inverted region appears to be translocated to a separate chromosome.

### Trait mapping

**Figure 4:**
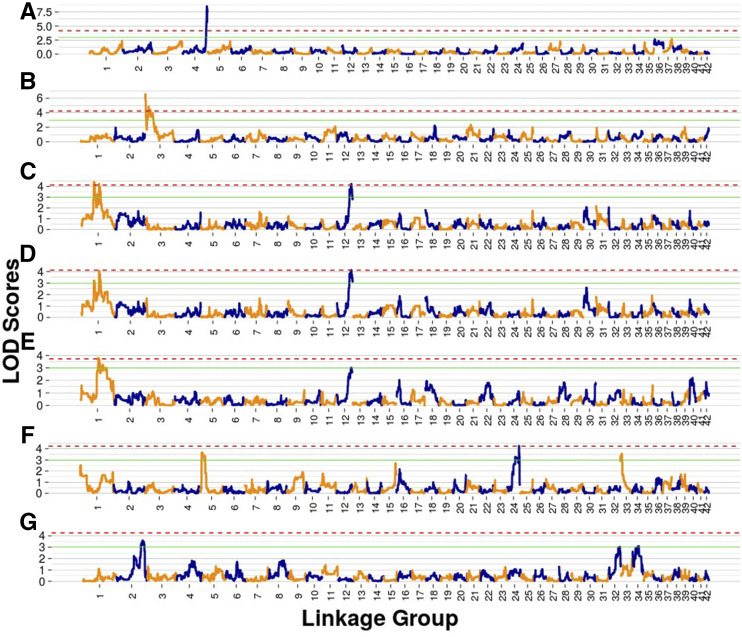
Panels display LOD values on the Y-axis *vs.* sex averaged map position (cM) for QTL scans for (A) the sex determination locus, (B) presence of spots and vermiculations, (C) fork length, (D) weight, (E) condition factor, (F) PCA1, and (G) PCA2. The dashed red line corresponds to the *P* < 0.05 significance threshold for LOD scores. The solid green line corresponds to the LOD threshold of 3 used to identify peaks putatively associated with each trait.

Multiple quantitative trait loci were detected for the evaluated traits (Table 5, Figure 4). A highly significant QTL for presence of spots and vermiculations mapped to a sex-averaged position of 3 cM on Sna3 (VPA1, 95% CI = 0-4.485 cM, LOD = 6.563, *P* = 0.001). We identified 16 candidate genes associated with this peak, including melanoregulin-like (*MREG-L*, Arctic char scaffold NW_019942894.1: 64734-79619; Sna3, 1.575 cM). A second QTL for this trait mapped 21.095 cM on the same linkage group (VPA2, 95% CI = 19.685 – 30.175, LOD = 4.850, *P* = 0.014). A total of 176 candidate genes were identified within this QTL credible interval. The four genes closest the highest LOD value were transcription factor 20-like (*TCF20-L*), retinoic acid induced 1-like (*RAI1-L*), sterol regulatory element binding factor 1-like (*SREBF1-L*), and calcium channel voltage-dependent T type alpha 1I subunit-like (*CACNA1I-L*; Table S3). These QTL explained 11.5 and 10.8% of phenotypic variance, respectively (Table 5).

**Table 5:** Linkage map positions (cM) of QTL peaks detected for the sex determination locus, presence-absence of vermiculations and spots, fork length, shape variable PCA1, shape variable PCA2, weight, and condition factor (Trait column). CI_Low and CI_High are the upper and lower bounds of the 95% credible interval for map positions for each QTL peak. LG is the linkage group on which the QTL was detected. Model lists the model used for QTL mapping in r/qtl2. Positions are sex averaged map positions. LOD scores are the differences in log10 likelihoods for models assuming presence or absence of a QTL at the locus in question (reported by r/qtl2). The estimates proportion of phenotypic variance explained by each QTL peak is listed in the PVE column. Estimated additive and dominant effects for the peak in question are also listed. P-values are those obtained via the permutation test described.

Trait	LG	Position (cM)	CI_Low	CI_High	Model	LOD	Additive Effect	Dominance Effect	PVE	P-value
Sex	Sna4	78.54	75.66	82.14	Binary	8.538	1.049	−0.045	0.115	<0.001***
Sex	Sna4	84.43	82.14	86.12	Binary	8.04	1.055	−0.044	0.108	<0.001***
Vermiculation	Sna3	3.00	0.00	4.49	Binary	6.563	1.595	0.278	0.191	0.001***
Vermiculation	Sna3	21.10	19.69	30.18	Binary	4.855	0.103	1.048	0.145	0.014*
Fork Length	Sna1	39.00	36.94	44.60	Normal	4.401	18.905	8.058	0.107	0.030*
Fork Length	Sna1	60.27	51.48	66.07	Normal	4.224	15.910	9.172	0.103	0.043*
Fork Length	Sna12	57.63	51.84	62.03	Normal	4.226	−11.693	10.910	0.103	0.043*
PCA1	Sna5	11.83	10.80	16.15	Normal	3.651	−0.005	0.011	0.111	0.156
PCA1	Sna24	35.99	27.30	44.50	Normal	4.259	−0.003	−0.011	0.128	0.049*
PCA1	Sna33	4.55	0.00	6.39	Normal	3.554	0.008	0.007	0.108	0.184
PCA2	Sna2	64.47	45.94	80.32	Normal	3.594	0.006	0.000	0.109	0.188
PCA2	Sna32	45.75	27.93	50.59	Normal	3.041	0.004	−0.001	0.093	0.451
PCA2	Sna34	22.82	0.00	39.41	Normal	3.087	0.005	0.000	0.095	0.423
Weight	Sna1	60.27	37.37	72.40	Normal	4.052	48.657	29.021	0.099	0.062
Weight	Sna12	57.67	50.55	64.15	Normal	4.13	−40.950	29.692	0.101	0.049*
Condition	Sna1	60.27	52.60	73.11	Normal	3.796	0.050	0.033	0.093	0.045*
Condition	Sna12	60.10	47.72	64.15	Normal	3.009	−0.053	0.001	0.074	0.278

Significant QTL for fork length mapped to two locations on Sna1 (FL1, 39.00 cM, 95% CI = 36.94 – 44.6cM, LOD = 4.401, *P* = 0.03, and FL2, 60.265 cM, 95% CI = 51.475 – 66.07 cM, LOD = 4.224, *P* = 0.043) and one location on Sna12 (FL3, 57.630 cM, 95% CI = 51.835-63.03 cM, LOD = 4.226, *P* = 0.043). A significant QTL for condition also mapped to 60.265 cM on Sna1 (CF1, 95% CI = 52.6 – 73.105, LOD = 3.796, *P* = 0.045) and a QTL for weight mapped to 57.665 cM on Sna12 (W1, 95% CI = 50.55 - 64.15 cM, LOD = 4.13, *P* = 0.045). Suggestive QTL (LOD > 3, *P* > 0.05) were detected on Sna1 (60.265 cM, 95% CI = 37.365 – 72.4, LOD = 4.052, *P* = 0.062) and Sna12 (60.095 cM, 95% CI = 47.72 – 64.15 cM, LOD = 0.009, *P* = 0.278) for weight and condition factor, respectively. We identified 39 candidate genes associated with peak FL1, 137 genes associated with FL2, and 77 genes associated with FL3. We did not search for candidate genes for other growth and body condition related QTL (weight and condition factor) because the locations and credible intervals for these QTL overlapped almost perfectly with those detected for fork length (Table 5).

Suggestive QTL were detected for the PCA1 body shape variable on Sna5 (11.830 cM, 95% CI = 10.8 – 16.145, LOD = 3.651, *P* = 0.156), Sna24 (PCA1_1, 35.990 cM, 95% CI = 27.3 – 44.5 cM, LOD = 4.259, *P* = 0.049), and Sna33 (4.550 cM, 95% CI = 0 – 6.39 cM, LOD = 3.554, *P* = 0.184); however only the peak on Sna24 was statistically significant. Suggestive QTL were detected for PCA2 on Sna2 (64.464 cM, 95% CI = 45.94 – 80.32, LOD = 3.594, *P* = 0.188), Sna32 (45.745 cM, 95% CI = 27.925 – 50.59 cM, LOD = 3.041, *P* = 0.451), and Sna34 (22.820 cM, 95% CI = 0 – 39.405 cM, LOD = 3.087, *P* = 0.423); however, none of these QTL were found to be statistically significant. 111 candidate genes were identified for the significant QTL interval for PCA1 on Sna24.

Our *sdY* presence-absence assay produced a male specific peak in melt curve derivative plots at approximately 84°. A melt curve derivative peak existed for *18S* at approximately 85°. Of the 16 known males used to validate the assay, 15 produced *sdY* peaks and 16 produced *18S* peaks. The 16 known females tested all yielded *18S* peaks; however, *sdY* peaks were absent in all females as expected. We were ultimately able to determine genotypic sex for 323 offspring produced from diploid crosses. Mapping *sdY* presence-absence as a binary trait using qtl2 identified strong peaks of association at 78.54 cM (95% CI = 75.66 – 82.14 cM, LOD = 8.538, *P* < 0.001) and 84.43 cM (95% CI = 82.14 – 86.12 cM, LOD = 8.04, *P* < 0.001) on Sna4.

### Recombination rates

Sex averaged recombination rates estimated by alignment to the Arctic char genome ranged from 0.138 to 2.935 cM/MB with a mean of 0.817 cM/MB (Table S4; SD = 0.537) across LGs. In general, recombination rate estimates generated by mapping to the Arctic char genome were lower than those obtained from mapping to rainbow trout or Atlantic salmon genomes (Table S4, Figure S9). Consistent with previous studies on salmonids, male recombination rates were considerably lower than those observed for females (Figure S9). For example, the mean male recombination rate base on alignment to the Rainbow Trout genome was 0.203 cM/MB (SD = 0.133), while the mean female recombination rate was 1.31 cM/MB (SD = 0.602). Alignment of male and female linkage maps to divergent reference genomes demonstrated that male recombination is highly suppressed except for near telomeres (Figures S1-S3).

## Discussion

### Map evaluation

Multiple lines of evidence suggest this linkage map provides an accurate representation of the lake trout genome. First, we identified a single centromere for each chromosome (except Sna42), suggesting that linkage groups were appropriately split. In all cases, centromere mapping locations derived from half-tetrad and RFm analysis either overlapped or were in close proximity (Figure 2, Table S2). For acrocentric and telocentric chromosomes, centromeres always mapped to the end of the chromosome with the highest female recombination rate and lowest male recombination rate, which matches results from previous centromere mapping efforts in salmonids suggesting that male recombination occurs almost exclusively near telomeres (Moen *et al.* 2008; Miller *et al.* 2012; McKinney *et al.* 2016). Second, our homology analysis demonstrated a high degree of contiguity between existing genome assemblies and the map presented here (Figures S1-S7). Finally, our sex determination locus mapping results are concordant with cytogenetic studies. Previous cytogenetic analysis of male and female lake trout identified sex-specific quinacrine and C-banding patterns on a large submetacentric chromosome (Phillips and Ihssen 1985). Mapping *sdY* presence-absence using the linkage map demonstrated with high certainty that the sex locus exists near one of the telomeres of Sna4 (Figure 4), which is metacentric or submetacentric based on RFm and half tetrad analysis (Figure 2). Although two significant peaks were detected on this LG, they were in close proximity and credible intervals were adjacent, suggesting that they likely represent a single peak of association. This study and others suggest that *Salvelinus* species, and salmonids in general, have highly variable sex chromosome configurations. Specifically, the brook trout sex determination locus maps to a region that is homologous to the Arctic char sex chromosome; however, it localizes to a different arm (Sutherland *et al.* 2017), while the lake trout sex chromosome identified here lacks homology with those of all species examined (Sal4p.1 – Nugent *et al.* 2017; BC35 – Sutherland *et al.* 2017; Ssa02, Ssa03, and Ssa06 – Kijas *et al.* 2018; Omy29 – Pearse *et al.* 2019). Many previous studies have identified variation in sex locus mapping position both within and between salmonid species (Woram *et al.* 2003; Lubieniecki *et al.* 2015; Sutherland *et al.* 2017; Kijas *et al.* 2018), even though the same gene ultimately underlies sex determination in most cases (Yano *et al.* 2013). Our results add to a growing body of literature suggesting that sdY is a conserved, yet highly mobile, sex determination gene in salmonids.

Furthermore, the lake trout linkage map presented here is of similar density to those used to scaffold genome assemblies for other salmonids (Christensen *et al.* 2018a, 2018b) and provides valuable information on the order of loci along chromosomes and recombination rates between loci. In general, male:female map length ratios and estimated sex averaged map lengths were highly similar to those observed for other salmonids. For instance, Leitwein *et al.* (2018) found that chromosome specific recombination rates varied from 0.21 – 4.1 cm/MB (mean =0.88) for brown trout, compared with 0.138 to 2.935 cM/MB (mean = 0.817) for lake trout based on mapping linkage mapped RAD loci to the Arctic char reference genome. Similar to other salmonids, we observed pronounced heterochiasmy, with male recombination being almost entirely suppressed except for near telomeres (Moen *et al.* 2004; Moen *et al.* 2008; McKinney *et al.* 2016; Leitwein *et al.* 2018)

### Structural variation

Combining centromere mapping locations with synteny analysis revealed that lake trout are differentiated from Arctic char, rainbow trout, and Atlantic salmon by multiple pericentric inversions, suggesting that centromeric instability (specifically on acrocentric and telocentic chromosomes) is potentially an important component of the salmonid evolutionary legacy (Table 4). Future work should evaluate whether these detected inversions are truly species diagnostic or if they are polymorphic within species. Large structural variants have previously been found to be associated with adaptive differentiation and life history variation within rainbow trout (Miller *et al.* 2012; Pearse *et al.* 2019) and inversions can contribute to pre or post-zygotic isolation between species or ecotypes (Kirkpatrick 2010). Future studies should evaluate the extent to which the structural variation detected here contribute to reproductive isolation and adaptive divergence within and between salmonid species. Sna24 presents one of the most striking examples of extensive structural variation in the genus *Salvelinus*, with multiple paracentric and pericentric inversions differentiating the lineages containing Arctic char and lake trout (Figure 3). With the exception of a putative inversion located between 0 and 12 cM, all other inversions on this LG were not observed in other salmonid species examined, suggesting that the other inversions on this chromosome (Sna24, Ssa14; Figure 3, column 2) are fixed or segregating within the Arctic char lineage or within the Salvelinus clade containing Arctic char, bull trout (*S. confluentus*), dolly varden trout (*S. malma*), and white char (*S. albus*). This hypothesis is supported by results from MapComp which suggested that brook trout, the most closely related extant species to lake trout (Crête-Lafrenière *et al.* 2012), and lake trout are not differentiated by any inversions on this linkage group. A large inversion spanning the centromere of Sna11 shows clear evidence of being differentially fixed between lake trout and all other salmonids except brook trout (Figure 3, Figures S1-S3, Figure S7). Inversions located on Sna10, Sna24, and Sna34 also appear to be differentially fixed between the lake trout – brook trout lineage and all other salmonids; however, interpretation is complicated by subsequent translocations and inversions that occurred in other taxa (Table 4). A large pericentric inversion on Sna12 appears to differentiate lake trout from all other salmonids, including brook trout (Figure S7). MapComp results also suggest that two large inversions on Sna28 (homologous to the brook trout sex chromosome - BC35; Sutherland *et al.* 2017) and Sna23 (BC25) differentiate lake trout from closely related brook trout (Figure S7). It is unclear if these structural variants are truly fixed between species, or if they might be polymorphic within lake trout or brook trout. The inversion polymorphisms identified above could be associated with chromosomal speciation within the genus *Salvelinus* or adaptive divergence within salmonid species and warrant further examination.

The majority of detected inversions differentiating lake trout from other salmonids are pericentric, which is not entirely unexpected. Repeat-rich eukaryotic centromeres often demonstrate exceptionally high rates of evolution (Henikoff *et al.* 2001) and are prone to chromosomal breakage and the accumulation of structural variation (Kalitsis and Choo 2012; Barra and Fachinetti 2018). Sutherland *et al.* (2016) also identified evidence for multiple inversions differentiating salmonid species, including one pericentric inversion differentiating pink, chum, and sockeye salmon from other salmonids.

Evidence for F2 inviability and reduced reproductive success between hybrids are widespread (Stebbins 1958), including for pairs of closely related species within the salmonid lineage (Renaut and Bernatchez 2011). Bull trout and brook trout, for instance, readily produce F1 offspring but F2 offspring are rarely observed (Leary *et al.* 1993). Hybrids between westslope cutthroat (*Oncorhynchus clarkii lewisi*) and rainbow trout are viable but have dramatic reductions in reproductive success (Muhlfeld *et al.* 2009). Future work should evaluate if instances of reduced fitness in inter or intraspecific salmonid hybrids might be linked to combined deleterious effects of recombination at multiple pericentrically inverted loci. It would also be interesting to ascertain whether centromeric regions tend to harbor signals of adaptive divergence between salmonid species and morphotypes. For example, Ellegren *et al.* (2012) found elevated levels of divergence between *Fidecula* flycatcher species near centromeres. Given the prevalence of pericentric inversions on acrocentric and telocentric chromosomes, we also might expect these loci to be associated with adaptive ecophenotypic radiations that have occurred within *Salvelinus* (Eshenroder 2008) and *Coregonus* (Lu and Bernatchez 1999).

### Genomic basis for adaptive traits

Suggestive QTL for traits that differentiate lake trout morphotypes were detected on multiple linkage groups. This supports the hypothesis proposed by Perreault-Payette *et al.* (2017) that ecophenotypic divergence in lake trout has a polygenic basis. Our results suggest that the presence or absence of spots and vermiculations is controlled by either one or two loci on the same arm of linkage group Sna3. A search for candidate genes within the QTL mapping intervals identified melanoregulin-like (*MREG-L*) as a potential causal locus. The homolog of this gene is involved in the transfer of melanosomes from melanocytes to keratinocytes (Wu *et al.* 2012), and appears to control the distribution of pigments within mice hair (O’Sullivan *et al.* 2004). Pigmentation polymorphisms are common in lake trout (Wilson and Mandrak 2004; Zimmerman *et al.* 2007) and other trout and char (Gomez-Uchida *et al*. 2008), although it is unclear if the genes identified here explain skin pigmentation variation in other species and populations. Skin pigmentation variation has been shown to be associated with depth of capture in multiple lake trout populations and is hypothesized to be adaptive in some environments (Protas and Patel 2008); however, it is also possible that the trait is simply linked with some other adaptive traits. Pigmentation patterns are often linked to variation in behavior, immune response, and energy homeostasis in vertebrates, likely owing to pleiotropic effects of melanocortins (Ducrest *et al.* 2008). Pigmentation traits have also been linked to stress response in rainbow trout, Atlantic salmon, and Arctic char (Hoglund *et al.* 2000; Kittilsen *et al.* 2009).

Suggestive QTL for the composite body shape variable with the highest explanatory power (PCA1) were detected on Sna5, Sna24, and Sna33. Interestingly, each of these chromosomes appear to have undergone structural reorganization in relatively recent evolutionary history, based on alignment to the Arctic char genome (Figure S1). Specifically, Sna24 and Sna33 are fused in Arctic char and Sna5 is split into two chromosomes. Sna24 in particular appears to have accumulated multiple large inversions that differentiate this linkage group from the homologous region of the syntenic Arctic char chromosome. A QTL for condition factor, which is closely related to body shape (Froese 2006), has been previously detected on the brook trout linkage group homologous to Sna33 (linkage group BC16, Sutherland *et al.* 2017). Additional mapping crosses, ideally generated using ancestral populations with highly differentiated body shapes (leans *vs.* siscowet or divergent hatchery strains for example) would be valuable for further validating the existence of QTL detected here and improving our understanding of the genetic basis for adaptive divergence within lake trout.

Growth and body condition related traits all have suggestive QTL on linkage groups Sna1 and Sna12, indicating that genes on these chromosomes likely harbor variation that underlies differences in growth between populations. Linkage group Sna12 also appears to harbor an inversion that differentiates lake trout from other salmonid species examined, including brook trout. A previous study identified a putative growth rate QTL on the brook trout linkage group homologous to Sna12 (Figure S7; Sutherland *et al.* 2017; BC9). The same study identified a stress response QTL, measured as change in blood cortisol levels following handling stress, in brook trout on the chromosome homologous to Sna1 (Sutherland *et al.* 2017, BC6). Increased cortisol levels have been found to be negatively corelated with growth and condition factor in other salmonids (Barton *et al.* 1987; Reinecke 2010), suggesting that variation observed in our families could actually be due to variation in stress response. There is evidence for variation in fitness among lake trout hatchery strains used to supplement and restore lake trout populations in the Great Lakes, with the strain from Seneca Lake, New York appearing to have a fitness advantage (Scribner *et al.* 2018). Great Lakes lake trout populations are heavily impacted by predation by invasive sea lamprey and previous work has shown that larger individuals have a greater probability of surviving lamprey attack (Swink 1990). Similarly, size-selective fisheries have also been shown to impose strong natural selection on growth in multiple species (Enberg *et al.* 2012). Future work could examine whether the chromosomal regions identified here are associated with size-at-age or are under selection in populations experiencing lamprey predation or size-selective fisheries.

Associations between environmental conditions and phenotype have been observed across the lake trout range and across salmonid species for the afore mentioned traits, suggesting adaptive significance in some contexts. For example, patterns and intensity of skin pigmentation, along with divergence in other traits, is commonly associated with depth-of-capture in both lake trout (Zimmerman *et al.* 2007; Marin *et al.* 2016) and Arctic char populations (Gomez-Uchida *et al.* 2008). Variation in skin pigmentation is potentially involved with predator avoidance and camouflage, feeding behavior, mate choice (Protas and Patel 2008), or protection from ultraviolet radiation (Yan *et al.* 2013). Differences in age specific growth rates are also frequently observed between humper, lean, and siscowet-like lake trout morphotypes (Burnham-Curtis and Bronte 1996; Hansen *et al.* 2012) — as well as between Arctic char morphotypes (Jonsson *et al.* 1988; Snorrason *et al.* 1994; Adams *et al.* 1998). These differences in growth rate likely reflect variation in allocation of resources toward growth and reproduction, adaptation to nutrient stress (Arendt 1997), or plastic responses to environmental variation (Hindar and Jonsson 1993).

Morphotypes can also often be differentiated based on body shape differences, which are hypothesized to be optimized for different feeding behaviors and modes of locomotion (Bond 1979; Muir *et al.* 2014; Perreault-Payette *et al.* 2017). For example, the streamlined body shape of leans has been hypothesized to be adaptive for swimming and predation in shallower nearshore environments (Bond 1979; Muir *et al.* 2014), while the more deep-bodied shape of siscowet lake trout is believed to reflect adaptation for vertical migration and foraging in deep-water habitats (Webb 1984; Muir *et al.* 2014). Morphotypes with traits reflecting those observed in the species native range have the potential to emerge rapidly in some introduced invasive populations (Stafford *et al.* 2013), suggesting a high degree of phenotypic plasticity or exceptionally strong selection favoring divergence.

Unfortunately, many lake trout metapopulations of conservation concern have experienced reductions in abundance and decreases in ecophenotypic diversity as a result of overexploitation and introduction of invasive species (Krueger and Ihssen 1995; Hansen 1999). For example, in the Great Lakes the siscowet morphotype has been extirpated from all lakes except Lake Superior (Krueger and Ihssen 1995). The results presented here enhance understanding of the genetic architecture of traits that underlie trophic specialization in lake trout and could aid in restoring genetic and phenotypic diversity in lakes where it has been lost.

## Conclusions

We identified multiple structural variants potentially involved in speciation and adaptation within the genus *Salvelinus*, mapped the lake trout sex determination locus, and identified QTL for traits believed to be adaptively significant in lake trout populations. Future work should use additional QTL mapping crosses and association studies in wild populations to evaluate if the QTL identified here are consistently associated with the phenotypic variation examined, as well as other phenotypes that differentiate lake trout morphotypes. Trophically specialized lake trout morphotypes and adaptively diverged populations are differentiated by multiple other traits (*i.e.*, tissue lipid content, fin size, diet; Thurston 1962; Eschmeyer and Phillips 1965; Zimmerman *et al.* 2006; Zimmerman *et al.* 2007). QTL mapping studies using later generation crosses or genome-wide association studies in wild populations would be particularly useful for fine-scale localization of genotype-phenotype associations within QTL credible intervals identified here. Additionally, QTL mapping efforts can yield different results for different families and the genetic basis for some traits often varies across populations (Santure *et al.* 2015). The lake trout linkage map will allow further examination of the genetic basis for ecophenotypic variation in lake trout and will enable additional exploration of chromosomal evolution within the genus *Salvelinus*. Perhaps most important, this resource will allow for the assembly of a chromosome-anchored reference genome for lake trout, which will greatly facilitate future genomic research on this important species.
